# Distinct choline metabolic profiles are associated with differences in gene expression for basal-like and luminal-like breast cancer xenograft models

**DOI:** 10.1186/1471-2407-10-433

**Published:** 2010-08-17

**Authors:** Siver A Moestue, Eldrid Borgan, Else M Huuse, Evita M Lindholm, Beathe Sitter, Anne-Lise Børresen-Dale, Olav Engebraaten, Gunhild M Mælandsmo, Ingrid S Gribbestad

**Affiliations:** 1 Department of Circulation and Medical Imaging, Norwegian University of Science and Technology (NTNU), Trondheim, Norway; 2 Department of Genetics, Institute for Cancer Research, Oslo University Hospital Radiumhospitalet, Oslo, Norway; 3 Department of Tumor Biology, Institute for Cancer Research, Oslo University Hospital Radiumhospitalet, Oslo, Norway; 4 Institute for Clinical Medicine, Faculty of Medicine, University of Oslo, Oslo, Norway

## Abstract

**Background:**

Increased concentrations of choline-containing compounds are frequently observed in breast carcinomas, and may serve as biomarkers for both diagnostic and treatment monitoring purposes. However, underlying mechanisms for the abnormal choline metabolism are poorly understood.

**Methods:**

The concentrations of choline-derived metabolites were determined in xenografted primary human breast carcinomas, representing basal-like and luminal-like subtypes. Quantification of metabolites in fresh frozen tissue was performed using high-resolution magic angle spinning magnetic resonance spectroscopy (HR MAS MRS).

The expression of genes involved in phosphatidylcholine (PtdCho) metabolism was retrieved from whole genome expression microarray analyses.

The metabolite profiles from xenografts were compared with profiles from human breast cancer, sampled from patients with estrogen/progesterone receptor positive (ER+/PgR+) or triple negative (ER-/PgR-/HER2-) breast cancer.

**Results:**

In basal-like xenografts, glycerophosphocholine (GPC) concentrations were higher than phosphocholine (PCho) concentrations, whereas this pattern was reversed in luminal-like xenografts. These differences may be explained by lower choline kinase (*CHKA*, *CHKB*) expression as well as higher PtdCho degradation mediated by higher expression of phospholipase A2 group 4A (*PLA2G4A*) and phospholipase B1 (*PLB1*) in the basal-like model. The glycine concentration was higher in the basal-like model. Although glycine could be derived from energy metabolism pathways, the gene expression data suggested a metabolic shift from PtdCho synthesis to glycine formation in basal-like xenografts. In agreement with results from the xenograft models, tissue samples from triple negative breast carcinomas had higher GPC/PCho ratio than samples from ER+/PgR+ carcinomas, suggesting that the choline metabolism in the experimental models is representative for luminal-like and basal-like human breast cancer.

**Conclusions:**

The differences in choline metabolite concentrations corresponded well with differences in gene expression, demonstrating distinct metabolic profiles in the xenograft models representing basal-like and luminal-like breast cancer. The same characteristics of choline metabolite profiles were also observed in patient material from ER+/PgR+ and triple-negative breast cancer, suggesting that the xenografts are relevant model systems for studies of choline metabolism in luminal-like and basal-like breast cancer.

## Background

Optimal treatment of individual breast cancer patients is still a major challenge in oncology. An approach to improve and individualize the treatment beyond the markers and stratification tools used at present, is through molecular subtyping of breast cancer [1]. Based on variation in gene expression profiles, five molecular subtypes have been identified [1-3]. The gene expression patterns of these subtypes are similar across multiple samples from the same tumor, shows no treatment-related changes and have been reproduced in a number of patient populations [2-7]. However, the current use of these molecular subgroups in clinical practice remains limited. Further understanding of the differences in biology between the various subtypes is needed in order to predict therapeutic response and provide individual treatment strategies based on gene expression profiles [8].

Elevated levels of choline metabolites is a known feature of breast cancer, and it has been shown that drugs targeting choline metabolism have selective *in vivo *and *in vitro *cytotoxic efficacy against a variety of cancer types [9-13]. Magnetic resonance spectroscopy (MRS) is a valuable tool for studies of choline metabolism both in patients and in experimental systems [14]. High resolution magic angle spinning (HR MAS) MRS of *ex vivo *tissue samples has been particularly useful, as it allows assessment of individual choline metabolites in intact tissue specimens. Increased concentrations of choline, phosphocholine (PCho) and glycerophosphocholine (GPC) has been demonstrated both in cultured breast cancer cells [15-17] and in human breast cancer biopsies [18-21]. It has also been shown that choline metabolism is altered following chemotherapy, suggesting the possibility of using MRS for therapy monitoring [22-24]. However, to utilize these findings in diagnosis and individualized therapy monitoring of breast cancer patients, a better understanding of the choline metabolism abnormalities on a molecular level is needed.

Several studies investigating expression of genes involved in metabolism of phosphatidylcholine (PtdCho) have been performed using breast cancer cell lines [16,17,25,26]. PtdCho is an important cellular membrane lipid, and this metabolic pathway directly involves choline, PCho and GPC. In addition, genes involved in transmembrane choline transport and conversion of choline to glycine have been suggested to be important for the observed choline concentrations in breast cancer cells [16,27]. The choline metabolism profiles observed in cultured breast cancer cells are more homogenous than those seen in human biopsies. In order to bridge the gap between *in vitro *research and clinical breast cancer, there is a great need for animal models representing different types of breast cancer for use in functional and mechanistic studies. Serial orthotopic transplantation of clinical tumor isolates in immunodeficient mice is considered a promising tool for investigation of human breast cancer biology [28]. Establishment of relevant experimental models of basal-like breast cancer is especially important both in order to understand the special characteristics of this subtype, to find potential new molecular targets for therapy and to establish potential biomarkers for monitoring response to therapy.

The aim of this study was to compare the choline metabolite patterns in animal models of basal-like and luminal-like subtypes of breast cancer, and to study the expression of genes related to choline metabolism in order to explain the differences between the two breast cancer subtypes. The two orthotopic xenograft models used, MAS98.12 and MAS98.06, represent basal-like and luminal-like subtypes of breast cancer, respectively [29]. Both models have been established by direct inoculation of primary human tumor material into immunodeficient animals. The content of creatine, choline, PCho, GPC, taurine and glycine in the xenografts as well as human breast cancer tissue samples was determined using HR MAS MRS. The molecular basis of the observed differences in choline metabolism was studied using gene expression microarray data. In order to evaluate if the xenograft models are representative for human disease, the metabolic profiles were compared to corresponding profiles from patients with ER+/PgR+ or triple negative breast cancer.

## Methods

### Animal model

The MAS98.12 and MAS98.06 tumor models were established by orthotopic implantation of biopsy tissues from primary mammary carcinomas in SCID mice as previously described [29]. Both the primary carcinomas and the xenograft models have been characterized using gene expression profiling. These analyses demonstrated that the primary carcinomas could be classified as luminal-like and basal-like subtypes of breast cancer, and that these molecular subtypes were retained in the MAS98.06 (luminal-like) and MAS98.12 (basal-like) xenografts. Relevant characteristics of the models are presented in Table 1. The tumors are serially transplanted. Tissue used for HR MAS MRS was from passage 47 (MAS98.12) and 28 (MAS98.06), and tissue used for RNA microarray analysis was from passage 45 (MAS98.12) and 25 (MAS98.06).

**Table 1:** Summary of xenograft characteristics

	Basal-like xenograft (MAS98.12)		Luminal-like xenograft (MAS98.06)	
	
	Primary tumor	Xenograft	Primary tumor	Xenograft
**Tumor grade**	Grade III IDC	NA	Grade III IDC	NA

**Lymph node status**	No metastasis	NA	Metastasis to 12 of 25 nodes	NA
			No distant metastases	

**Differentiation**	Poorly differentiated	Poorly differentiated	Well differentiated	Poorly differentiated

**Hormone receptor status**	ER-/PgR+**	ER-/PgR-	ER+/PgR+	ER+/PgR+

ERBB2 amplification*	Negative	Negative	Negative	Negative

**Intrinsic molecular subtype**	Basal-like	Basal-like	Luminal-like	Luminal-like

**TP53 status**	Wildtype	Mutated	Mutated	Mutated

**Volume doubling time**	NA	1-2 days	NA	7 days

**Proliferation index (Ki67)**	Missing	28%	Missing	35%

Summary of characteristics related to genotype and phenotype of the xenograft models

* Measured at the DNA level by array Comparative Genomic Hybridization (aCGH)

** The primary basal-like carcinoma had very weak cytoplasmic staining for PgR^29^.

The animals were kept under pathogen-free conditions. Housing conditions included temperature between 19°C and 22°C, humidity between 50% and 60%, 20 air changes/hr and a 12 hr light/dark cycle. The animals were fed RM1 diet (Scanbur BK, Norway) and distilled tap water *ad libitum*. The drinking water was supplemented with 17-β-estradiol at a concentration of 4 μg/ml in order to ensure stimulation of the estrogen receptors and promote tumor growth in the MAS98.06 xenografts. With respect to tumor growth rate, this estrogen supplement correspond to the use of s.c. continuous release 17-β-estradiol pellets (1.7 mg/pellet), which were used during establishment of the animal models [29]. To provide equal experimental conditions, the MAS98.12 xenografts also received estradiol supplement. This could in theory cause non-ER-mediated effects on choline metabolite profile. However, the similarities between human tissue samples and xenograft tissue suggest that such effects are insignificant in ER-breast cancer.

Following sacrifice by cervical dislocation, tumor tissue was harvested from 10 animals from each model for the HR MAS MRS analyses and for 6 animals from each model for gene expression microarray analyses, at tumor diameters of approximately 13-15 mm. Samples were put in cryogenic vials and immersed in liquid nitrogen immediately after dissection and stored under cryogenic conditions until analysis. All procedures and experiments involving animals were approved by The National Animal Research Authority, and carried out according to the European Convention for the Protection of Vertebrates used for Scientific Purposes.

### Human tissue samples

For comparison of xenograft models with human breast cancer tissue, biopsies from 22 breast cancer patients were identified in our internal database based on histopathology/immunohistochemistry data. Patients with either ER+/PgR+ (n = 14) or triple negative (n = 8) phenotype were included. Biopsy material was obtained during surgery and immediately frozen in liquid nitrogen. Histopathology and immunohistochemistry data for the selected patients was obtained from hospital records. Patient and tumor characteristics are presented in Table 2. The biopsy material was subject to HR MAS MRS analysis and subsequent histopathological evaluation using hematoxylin/eosin (HE) staining. The use of patient material was approved by the Regional Committee for Medical and Health Research Ethics, and informed written consent was obtained from all included patients.

**Table 2:** Summary of patient characteristics

Subtype	n	Patient age (years)	Phenotype	Tumor grade1/2/3	Tumor size (cm)	Mean tumor fraction (%)	Mean connective tissue fraction (%)	Mean fatty tissue fraction (%)
ER+/PgR+	14	57 ± 16	ER+/PgR+	1/10/3	2.3 ± 1.3	23 ± 11	72 ± 11	5 ± 7

Triple negative	8	57 ± 17	ER-/PgR-/HER2-	0/3/5	2.2 ± 1.0	38 ± 32	55 ± 31	6 ± 7

Summary of patient and sample characteristics of the different subgroups of human tissue samples (mean ± SD)

### HR MAS MRS of xenograft tissue

Storage time before HR MAS MRS analysis was less than one month for all 20 samples. Macroscopically viable tumor tissue was cut to fit a 30 μl disposable insert (Bruker Biospin Corp.), prefilled with 3 μl PBS made on D_2_O containing 98.8 mM trimethylsilyltetradeuteropropionic acid (TSP) for chemical shift referencing. The average sample weight was 15 ± 3 mg (mean ± SD). HR MAS MR spectra were recorded using a Bruker AVANCE DRX600 spectrometer equipped with a ^1^H/^13^C HR MAS probe (Bruker BioSpin Corp.). Samples were spun at 5 kHz with an instrumental temperature setting of 4°C. A pulse-acquired experiment including the ERETIC sequence (ereticpr.drx; Bruker) was performed for all samples. The ERETIC signal was positioned at -1.0 ppm. The water resonance was saturated for 15 seconds (60 dB continuous wave), followed by a 60-degree pulse for excitation. Signals were collected over a sweep width of 16.7 ppm. 128 FIDs were acquired into 64K points during 3.28 seconds. Spectra were Fourier transformed into 128K after 0.3 Hz exponential line broadening and chemical shifts were calibrated to the TSP singlet at 0 ppm. Spectral assignments were performed based on a previous HR MAS MRS study of breast cancer lesions [30]. One HR MAS MRS spectrum from the MAS98.06 animals was lost due to technical error.

The regions from 0.20 to -0.20 ppm (TSP), -0.85 to -1.15 ppm (ERETIC) and 3.60 to 2.90 ppm (glycine, taurine, GPC, PCho, choline, and creatine) were selected for quantification in all spectra. Peak areas were calculated by curve fitting (PeakFit v 4, Systat Software Inc) using a combination of Gaussian and Lorentzian line-shapes (Voigt function). The correlation coefficient of the fit (r^2^) was > 0.95 for all spectra. The ERETIC signal was quantified to 3.17 × 10^-7 ^moles using a series of creatine calibration standards as previously described [31]. Concentrations of tissue metabolites ([*MET*]) were calculated relative to the ERETIC signal using equation (1):

(1) $$[MET]=\frac{A_{MET}}{A_{ERETIC}}\times \frac{1}{k_{MET}}\times \frac{n_{ERETIC}}{m_{sample}}$$

A_*MET *_and A_*ERETIC *_are the calculated areas of the metabolite and the ERETIC signals, respectively; *k*_*MET *_is the number of protons giving rise to the metabolite signal; *n*_*ERETIC *_is the number of moles the ERETIC signal represents; and *m*_*sample *_is the mass of the sample. The metabolite concentrations measured using the ERETIC signal were compared using a 2-sided Student's t-test with a significance level of p < 0.05 using Sigmaplot 11.0 (Systat Software Inc.).

### HR MAS MRS of human tissue samples

Human tissue samples were prepared for HR MAS MRS analysis using the same procedure as the xenograft samples. Spectra were acquired using a spin-echo Carr-Purcell-Meiboom-Gill sequence (cpmgpr; Bruker) with 2 s water suppression prior to a 90° excitation pulse. The spin-echo sequence for suppression of broad peaks was performed using a delay of 1 ms repeated 136 times, resulting in an effective echo time of 285 ms. A total of 128 scans over a spectral region of 10 kHz were collected into 32k points during 1.64 s. The spectra were Fourier transformed into 128 K after 0.3 Hz exponential line broadening, and the metabolite region from 3.60 to 3.00 ppm was selected for further evaluation. The spectra were normalized by scaling the spectral data of all samples to achieve an equal total area for each spectrum. Metabolite peak areas were then obtained by curve fitting as described above.

### Histopathology

Following HR MAS MRS analysis, the xenograft samples were fixed in 10% neutral buffered formalin and embedded in paraffin. One histopathological section were prepared from each sample, stained with hematoxylin/eosin/saffron (HES) according to standard protocol and evaluated microscopically. A visual evaluation with respect to the presence of viable tumor tissue and the extent of necrosis was performed. Tumor grade, hormone receptor status and HER2 expression of human tissue samples was obtained from hospital records. In addition, specimens analysed by HR MAS MRS were HES-stained and the relative areas of normal and neoplastic epithelial tissue, necrotic tissue, fat and fibrous connective tissue were scored.

### Gene expression analysis

Gene expression analysis was performed on tumor tissue from 6 animals from each of the two xenograft models, using a one-color microarray-based platform (Agilent). Total RNA was isolated from snap frozen tumor tissue using TRIzol (Invitrogen) and resuspended in RNase-free water. Total RNA (700 ng) was amplified, labelled with Cy3, and 1.65 μg cRNA was hybridized to 4 × 44 k Agilent Whole Human Genome Oligo Microarrays at 60°C and 10 rpm for 17 hours, according to the manufacturer's protocol. The arrays were scanned using an Agilent G2565A DNA microarray scanner and extracted using Feature Extraction (v 10.1.1.1, Agilent). One microarray from the MAS98.06 model was removed due to poor array quality. The microarray data was normalized and analysed using R (v 2.9.0) and the LIMMA Bioconductor package [32]. The raw signals were corrected for multiplicative detrending effects and the arrays were quantile normalized and log2 transformed. Probes which were flagged as outliers by the Feature Extraction software or were present in less than 30% of the samples, were removed. The signal intensities were averaged between duplicate probes, and the probe with the highest interquartile range was selected to represent each unique transcript.

A total of 119 genes were selected for further analysis. The selection criteria were a) genes involved in KEGG *homo sapiens *glycerophospholipid pathway hsa:00564 [33], or b) genes coding for proteins reported to be directly involved in choline transport and choline and glycine metabolism [16,34-36]. Of the selected genes, 117 were represented on the microarray (full gene list supplied as additional file 1).

Testing for differential expression between the xenograft models was performed using t-tests with Empirical Bayesian correction of the test statistics [32]. To account for multiple testing, an adjusted p-value of 0.05 (using Benjamini & Hochberg's false discovery rate) was defined as the threshold for significant differential expression between the xenograft models. The microarray data from the significantly differentially expressed genes was centered across genes and clustered across genes and samples using hierarchical clustering with Euclidian distance and complete linkage. The relationship between gene expression and metabolite concentrations was explored using Ingenuity Pathways Analysis (Ingenuity Systems), and an illustration was adapted from the canonical Glycerophospholipid Metabolism and Glycine, Serine and Threonine Metabolism pathways [33]. The abovementioned gene list was also extracted from microarray data from previously described passages of the same xenograft models [29], to ensure that gene expression remain stable throughout serial transplantation of the xenografts.

## Results

### Histopathology

All xenograft samples were found to contain mainly viable tumor tissue and stromal connective tissue, shown previously to be recruited mouse stromal tissue [29], with negligible necrosis (< 10% area) in 18 of 19 samples. The HR MAS MRS data was therefore considered to be representative of the metabolite concentrations in the solid tumors. One sample in the MAS98.06 group contained a necrotic area, microscopically estimated to 25% area of the specimen. However, the metabolite concentrations measured in this sample differed from the group mean by less than ± 2 SD, and the sample was therefore not excluded from the data set. The mean fractions of tumor and connective tissue in the human tissue samples are presented in Table 2.

### HR MAS MRS of xenograft samples

**Figure 1:**
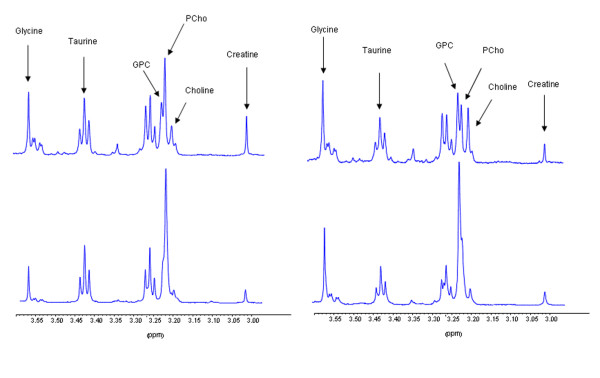
**Mean HR MAS MRS spectra from human tissue samples and xenograft tissue**. HR MAS MRS spectra (spectral region 3.6 to 3.0 ppm) of ER+/PgR+ (top left) and triple negative (top right) human tissue samples, and luminal-like (bottom left) and basal-like (bottom right) xenograft samples. Spectral assignments are provided for peaks used in quantification.

The HR MAS MRS analyses revealed several significant differences in the metabolic profiles of the two xenograft models. Mean ^1^H HR MAS MRS spectra from the two models are shown in Figure 1 (spectral region 3.6 - 3.0 ppm). The metabolites assigned in Figure 1 were quantifiable in all spectra. The metabolite concentrations calculated using the ERETIC reference signal are presented in Table 3. There was no significant difference in choline concentration between the models. However, the concentrations of GPC and PCho were significantly higher than the choline concentration in both the basal-like and the luminal-like model. While all the samples from basal-like xenografts showed higher concentration of GPC than PCho, the samples from luminal-like xenografts invariably showed lower concentrations of GPC than PCho. The differences in GPC and PCho concentrations between the two xenograft models were statistically significant (p < 0.001 and p < 0.01, respectively). The concentration of glycine was significantly higher in the basal-like than in the luminal-like model (p < 0.002).

**Table 3:** Metabolite concentrations

	MAS98.12 (n = 10)	MAS98.06 (n = 9)
Creatine	4.1 ± 1.4	3.4 ± 1.7
Choline	1.2 ± 0.7	0.9 ± 0.6
Phosphocholine *	4.5 ± 2.1	9.1 ± 4.4
Glycerophosphocholine **	9.8 ± 2.5	2.7 ± 1.7
Taurine	14.7 ± 4.1	19.1 ± 9.1
Glycine *	8.2 ± 3.0	4.0 ± 1.8

Metabolite concentrations in basal-like (MAS98.12) and luminal-like (MAS98.06) xenografts calculated from HR MAS MRS spectra using the ERETIC method (μmol/g, mean ± SD, * p < 0.01, ** p < 0.001)

### HR MAS MRS of human tissue samples

The HR MAS MRS spectra from the tissue samples were retrieved from our internal database, and mean spectra from the two groups are shown in Figure 1. The mean metabolite profiles demonstrated that triple negative breast cancer tissue had high GPC and low PCho concentrations, whereas tissue from ER+/PgR+ patients had low GPC and high PCho. There was a significant difference in the GPC/PCho peak area ratio between ER+/PgR+ and triple negative samples (0.8 ± 0.5 and 1.5 ± 0.7, respectively. p = 0.01), corresponding to the findings from the xenograft models. The mean spectra from human tissue samples also suggested that the glycine concentration was higher in triple negative breast cancer tissue samples. Using the glycine/total peak area ratio as marker for glycine content, this trend was not statistically significant (p = 0.19). The relative choline peak area was significantly higher in triple negative tissue (p < 0.00003) and the relative creatine peak area was significantly lower (p = 0.024).

### Gene expression analysis of xenograft tissue

**Figure 2:**
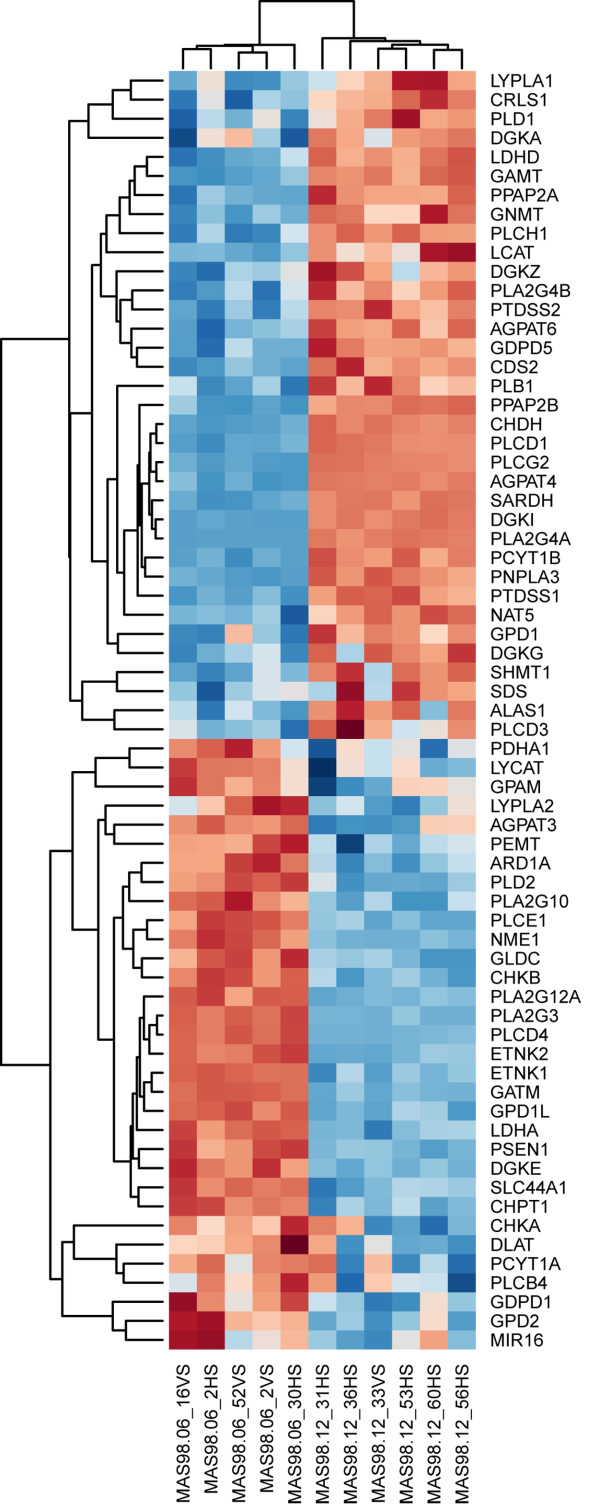
**Heatmap of differentially expressed genes in xenograft models**. Hierarchical clustering of the 67 differentially expressed genes (false discovery rate < 0.05) involved in the KEGG *homo sapiens *glycerophospholipid pathway hsa:00564, choline transport or directly involved in conversion from choline to glycine (Red: high expression compared to mean expression in xenograft samples. Blue: low expression compared to mean expression in xenograft samples). The microarray data was centred across genes and clustered across genes and samples using Euclidian distance and complete linkage.

Of the 119 investigated genes, 67 were differentially expressed between the xenograft models at a 5% adjusted (false discovery rate) significance level. Microarray data from earlier passages of the same xenograft models [29] showed similar trends of differential expression (data not shown). The complete results from the gene expression analysis are available as additional file. A heatmap of the differentially expressed genes is presented in Figure 2, with hierarchical clustering of genes and samples. In the following sections, only genes directly involved in synthesis and degradation of PtdCho from choline are considered.

Among the five selected genes coding for proteins known to be involved in transmembrane choline transport, only solute carrier family 44, member 1 (*SLC44A1*) showed significantly different expression between the two models. The expression of this transporter, also known as choline transporter-like protein 1 (*CTL1*), was lower in basal-like than luminal-like xenografts. Solute carrier family 22, member 1 (*SLC22A1*) and solute carrier family 44, member 2 (*SLC44A2*) were similarly expressed in the two models, whereas solute carrier family 5 (choline transporter), member 7 (*SLC5A7*) and solute carrier family 22 (organic cation transporter), member 2 (*SLC22A2*), were expressed below the limit of detection. *SLC5A7 *is also known as choline transporter 1 (*CHT1*), a high-affinity choline-specific transporter protein, whereas *SLC44A2 *is also known as choline-transporter like protein 2 (*CTL2*).

**Figure 3:**
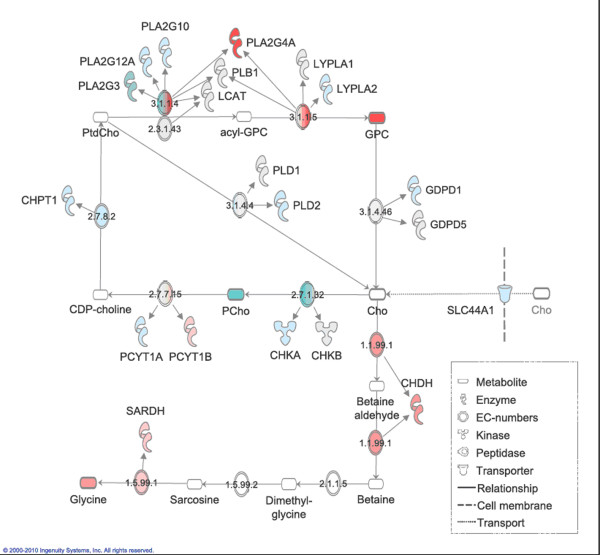
**Differences in gene expression and metabolite concentrations**. Differences in intracellular choline metabolism, choline transport and glycine formation between basal-like and luminal-like xenografts. Red: higher gene expression/metabolite concentration in basal-like xenografts. Blue: higher gene expression/metabolite concentration in luminal-like xenografts. White: Non-significant difference in expression/concentration. Log2-fold difference between models is indicated by color intensity. Only significantly differentially expressed genes are illustrated at each EC number, which represents specific enzymatic reactions. The metabolites which were measured with HR MAS MRS are outlined with a thicker line.

Genes directly involved in choline metabolism which were differentially expressed between the xenograft models are listed in Table 4 and 5. A schematic overview of intracellular choline metabolite concentrations and the comparative gene expression (the anabolic Kennedy pathway, PtdCho breakdown and conversion of choline to glycine) between the xenograft models is presented in Figure 3. As shown in Figure 3, choline is converted to PCho through the action of two isoforms of the same enzyme, choline kinase alpha and beta. The expression of the genes (*CHKA*, *CHKB*) coding for both isoforms was significantly lower in the basal-like than in the luminal-like model.

**Table 4:** Differentially expressed genes

Entrez ID	Probe name	Gene name	Encoded protein	Log2-fold difference	Adjusted p-value (false discovery rate)
5321	A_23_P11685	PLA2G4A	Phospholipase A2, group IV A	6.4	4.4E-^16^

55349	A_23_P69293	CHDH	Choline dehydrogenase	3.3	4.0E^-13^

1757	A_24_P35400	SARDH	Sarcosine dehydrogenase	2.5	7.6E^-12^

9468	A_24_P941353	PCYT1B	Phosphate cytidylyltransferase 1, choline, beta	1.7	3.7E^-9^

31896	A_23_P87401	GDPD5	Glycerophosphodiester phosphodiesterase domain containing 5	1.0	4.2E^-6^

8681	A_23_P403424	PLA2G4B	Phospholipase A2, group IV B	0.9	9.8E^-5^

9791	A_23_P168868	PTDSS1	Phosphatidylserine synthase I	0.9	9.6E^-7^

10434	A_23_P19192	LYPLA1	Lysophospholipase 1	0.9	0.001

3931	A_23_P218237	LCAT	Lecithin-cholesterol acyltransferase	0.8	0.0002

151056	A_23_P56356	PLB1	Phospholipase B1	0.7	0.0001

5337	A_23_P155335	PLD1	Phospholipase D1	0.7	0.0005

Genes directly involved in choline metabolism with significantly higher expression in basal-like (MAS98.12) than luminal-like (MAS98.06) tumors

**Table 5:** Differentially expressed genes

Entrez ID	Probe name	Gene name	Encoded protein	Log2-fold difference	Adjusted p-value (false discovery rate)
50487	A_23_P17814	PLA2G3	Phospholipase A2, group III	-3.1	1.1E-^12^

81579	A_23_P30020	PLA2G12A	Phospholipase A2, group XII A	-1.6	2.5E^-9^

56994	A_23_P105571	CHPT1	Choline phosphotransferase 1	-1.4	1.6E^-7^

23446	A_23_P216630	SLC44A1	Solute carrier family 44, member 1 (CTL1)	-1.1	7.9E^-7^

5338	A_23_P4308	PLD2	Phospholipase D2	-1.1	1.2E^-6^

8399	A_23_P88767	PLA2G10	Phospholipase A2, group X	-1.1	3.1E^-6^

1119	A_23_P314120	CHKB	Choline kinase beta	-0.8	2.0E^-6^

11313	A_24_P276490	LYPLA2	Lysophospholipase II	-0.4	0.005

5130	A_23_P252681	PCYT1A	Phosphate cytidylyltransferase 1, choline, alpha	-0.4	0.035

24657	A_23_P84666	GDPD1	Glycerophosphodiester phosphodiesterase domain containing I	-0.4	0.005

1119	A_23_P124742	CHKA	Choline kinase, alpha	-0.3	0.047

Genes directly involved in choline metabolism with significantly lower expression in basal-like (MAS98.12) than luminal-like (MAS98.06) tumors

Conversion of PCho to CDP-choline is mediated through the alpha and beta isoforms of phosphate cytidylyl transferase 1 (*PCYT1A *and *PCYT1B*). The expression of *PCYT1B *was significantly higher, and *PCYT1A *was significantly lower in the basal-like than the luminal-like model. The gene coding for choline phosphotransferase 1 (*CHPT1*), which converts CDP-PCho to PtdCho, had a significantly lower expression level in the basal-like than in the luminal-like model.

PtdCho is degraded by several different phospholipases. Enzymes in the phosphoplipase A2 group (*PLA2*) convert PtdCho to acyl-GPC. Of the 13 *PLA2 *isoforms studied, two were significantly higher expressed in the basal-like model, three were significantly lower expressed, five showed no significant difference in expression and three were below the limit of detection. The largest difference in gene expression between the two models was found for *PLA2G4A*, where a log2-fold difference of 6.4 in gene expression was observed. Phospholipase B1 (*PLB1*) is involved in both deacetylation steps from PtdCho to GPC, and was significantly higher expressed in the basal-like than in the luminal-like model.

Phospholipase D, with the two isoforms *PLD1 *and *PLD2*, converts PtdCho to choline. The expression of *PLD1 *was significantly higher and the expression of *PLD2 *was significantly lower in the basal-like compared to the luminal-like model. Other genes related to the degradation of PtdCho, such as lecithin-cholesterol acetyltransferase (*LCAT*) and phosphatidylserine synthase 1 (*PTDSS1*) also had significantly higher expression levels in the basal-like than in the luminal-like model. The *GDPD5 *gene, coding for glycerophosphodiester phosphodiesterase (GDPD) was significantly higher expressed in the basal-like model than in the luminal-like model, indicating that GPC degradation may occur at a higher rate in basal-like xenografts. However, an isoform of this gene, *GDPD1*, was higher expressed in the luminal-like xenografts. As shown in Figure 3, choline dehydrogenase (*CHDH*) mediates the irreversible conversion of choline to betaine, which is a key precursor in the synthesis of glycine. The expression of *CHDH *was significantly higher in the basal-like than in the luminal-like model. Sarcosine dehydrogenase (*SARDH*), involved in the conversion of betaine to glycine, also had significantly higher expression levels in the basal-like model.

## Discussion

The HR MAS MRS data demonstrated significant differences in choline metabolite pattern between the basal-like and luminal-like xenograft models. In particular, the difference in GPC and PCho concentrations is an interesting finding, as the pattern seen in the basal-like model does not correspond to typical *in vitro *choline metabolite patterns [15,17]. In addition, expression data showed that several genes directly associated with choline metabolism differed significantly between the two models. Differences in expression of genes involved in choline metabolism corresponded to differences in metabolite concentrations, suggesting that transcriptional differences between the models are reflected in the HR MAS MRS spectra. The relative amounts of GPC and PCho in human tissue samples from triple negative and ER+/PgR+ subtypes of breast cancer corresponded well with the data from the xenografts.

In order to evaluate if the choline metabolism in the xenograft models is representative for basal-like and luminal-like breast cancer in humans, they were compared to data from triple negative and ER+/PgR+ breast cancer patients. It is assumed that the triple-negative phenotype is a valid surrogate marker for basal-like breast cancer, as approximately 90% of triple-negative breast carcinomas can be classified as basal-like based on the intrinsic molecular subtyping developed by Sørlie *et al *[3,37]. On the other hand, expression of estrogen and/or progesterone receptors is a typical feature of luminal A and B subtypes, whereas the ERBB2 and basal-like subtypes of breast cancer rarely express hormone receptors [38,39]. Therefore, the ER+/PgR+ phenotype is considered to be a valid surrogate marker for luminal-like subtypes of breast cancer.

Using gene expression profiling, the molecular causes for the differences in choline metabolism was further explored in the xenograft models. The heatmap of all 64 significantly differentially expressed genes in Figure 2, clearly shows that different sets of genes related to phospholipid metabolism are higher expressed the basal-like model compared to the luminal-like models. This indicates that the regulation of choline metabolism differ between the two xenograft models. Although this study does not provide data on metabolic flux, the methods used are suitable for highlighting key steps in choline metabolism. Comparison of these two disease models does not, however, give any information with respect to the difference between choline metabolism in normal breast versus breast cancer tissue. Nevertheless, gene expression profiling of the xenograft models showed significant differences in the expression of genes directly involved in choline metabolism, suggesting that these genes may play key roles in regulation of choline metabolite concentrations in human breast cancer.

Increased choline transport has been associated with the abnormally high concentrations of PCho observed in breast cancer [16,27,40]. In our study, the influx of choline in the two models could not be fully evaluated from the gene expression data, as only one of five investigated choline transporters was differentially expressed. Choline transport has been shown to be less important than PtdCho turnover for total choline metabolite concentrations [17]. Differences in choline uptake may still have impact on the choline metabolite concentrations, and specific studies using isotopically labelled choline could possibly allow accurate evaluation of choline transport rate in the two xenograft models.

In breast cancer cells, the intracellular metabolism of choline is divided in two major pathways as shown in Figure 3: Betaine production or PtdCho synthesis [27,34]. In the betaine synthesis pathway, choline is oxidized to betaine through the action of choline dehydrogenase (*CHDH*). Betaine is then demethylated to glycine. *In vitro *studies of MCF7-cells have shown that PtdCho synthesis is the pathway predominantly accountable for choline turnover [34]. The first step in the PtdCho synthesis pathway is the phosphorylation of choline through choline kinase, yielding PCho (Figure 3). It has been shown that increased expression of *CHKA *is critical for proliferation both of mammary epithelial cells and breast cancer [41], but *in vitro *studies of different breast cancer cell lines have not conclusively demonstrated a correlation between *CHKA *expression and PCho concentration [16,26]. In our study, the expression of *CHKA *and *CHKB *was significantly lower in the basal-like than in the luminal-like model, although some variability in expression was observed (Figure 2). This is consistent with the lower PCho concentrations measured in the basal-like model. Betaine production is thought to contribute only slightly to the overall conversion of choline, and neither choline transport nor GPC degradation is conclusive with respect to their contribution to the choline pool. As normal breast tissue or benign breast lesions rarely exhibit increased choline metabolite levels, the xenograft models are believed to represent typical choline metabolism abnormalities of breast carcinomas [42,43]. Therefore, it should be stressed that *CHKA *and *CHKB *expression is likely to be upregulated in both xenograft models compared to normal breast tissue. The lower PCho concentrations in the basal-like xenografts may also in part be a result of higher *CHDH *expression. This suggests that conversion of choline to betaine is upregulated, shifting the metabolic flux in favour of glycine formation. *SARDH*, related to conversion of betaine to glycine, was also significantly higher expressed in the basal-like model. The concentration of glycine in the basal-like model was indeed higher than in the luminal-like model, suggesting that there is a difference in choline routing and glycine production between the two breast cancer subtypes. An association between tumor aggressiveness and glycine concentration has been noted also in clinical breast cancer tissue biopsies [21]. Abnormalities in cancer energy metabolism are widely recognized, and differences in glycine concentration between the two xenograft models in this study could well be an indirect result of this phenomenon.

Degradation of PtdCho is the primary source of GPC. The expression of *PLA2G4A*, *PLA2G4B*, *LCAT*, *LYPLA1 *and *PLB1*, which all are associated with this pathway, was higher in the basal-like model. Other genes (*PLA2G3*, *PLA2G12A*, *PLA2G10*, *LYPLA2*) were lower expressed in the basal-like model, and a clear association between PtdCho degradation and GPC concentration could not be concluded. However, *in vitro *studies have suggested that GPC concentrations are associated with *PLA2G4A *levels, which is consistent with our findings [17]. A lower rate of GPC degradation could account for the higher GPC concentration observed in the basal-like xenograft model. The expression of *GDPD5 *was, however, higher in basal-like xenografts. The observed differences between the two models in the relative expression of different genes assigned to the abovementioned enzymatic steps could be reflecting the relative importance of different gene products coding for proteins with the same enzymatic activity in the two models.

By associating choline metabolite concentrations with tumor cell phenotype, it has been proposed that PCho concentration increase with the malignancy of the tumor cell line when grown in culture [15]. However, other *in vitro *studies have failed to show a correlation between malignancy and choline metabolite concentrations [16]. It has been suggested that differences in experimental design, particularly the stage of cell growth, are accountable for these discrepancies [26]. In all the abovementioned *in vitro *studies of breast cancer cells, PCho concentrations were significantly higher than GPC concentrations. However, both in xenograft models of breast cancer and in clinical tissue samples, GPC concentrations higher than PCho concentrations have been observed [21,44]. GPC concentration has been shown to be negatively correlated with estrogen receptor content in breast carcinomas, which agrees with the relatively high GPC content in the basal-like xenograft [45]. Our data show that GPC concentration is significantly lower and PCho concentration is significantly higher in the luminal-like animal model, which represents a less aggressive disease than the basal-like model. This suggests that the relationship between choline metabolite concentrations and malignancy of solid tumors is more complex than indicated by studies of breast cancer cell lines. Discrepancies between *in vitro *data and clinical data may be attributed to the microenvironment of solid tumors. It has recently been shown that the metabolic profiles change when the same breast cancer cell lines are studied both *in vitro *and *in vivo *[46]. In addition, *in vitro *simulation of microenvironmental factors in solid tumors has demonstrated that PCho and GPC concentrations respond to changes in acidity, oxygenation level and glucose accessibility [44].

The relevance of the basal-like and luminal-like xenografts used in this study was further supported by comparing the choline metabolite pattern with that of human tissue samples from ER+/PgR+ and triple negative breast cancer. Evaluation of metabolite levels through relative peak areas demonstrated that the mean GPC/PCho ratio was significantly higher in triple negative breast cancer than in ER+/PgR+ breast cancer. The relative PCho area was significantly higher in ER+/PgR+ samples than in samples from triple negative breast cancer. A trend towards higher glycine concentration was also found in triple negative tissue samples. Interestingly, the choline concentration in triple negative breast cancer was higher than in ER+/PgR+ breast cancer. Overall, the striking similarity between xenografts and human tissue samples with respect to GPC and PCho levels suggest that the xenografts have maintained genetic and/or microenvironmental features from the primary carcinomas which are relevant for the choline metabolite pattern. The spectra from human tissue samples also suggest that PCho concentrations alone are not a reliable prognostic biomarker. The triple negative samples represent disease with poor prognosis, yet the PCho level in these samples appear to be significantly lower than in ER+/PgR+ samples. This finding encourages large-scale studies of the metabolite pattern and gene expression in the different molecular subtypes of breast cancer, as this may reveal new drug targets or suggest strategies for individualised therapy using drugs targeting the choline metabolism pathways.

When interpreting the gene expression data from the two xenograft models, it should be kept in mind that gene expression not always represents the actual enzymatic activity. Isoforms of the same enzyme may exhibit differences in transcriptional regulation, and mRNA concentrations do not account for post-translational modification of enzymes. In addition, the concentrations of all investigated choline-containing compounds are determined by more than one metabolic reaction. Thus, a simplistic model for correlating gene expression with metabolite concentration is not applicable. The net rate of all relevant metabolic reactions governs the metabolite concentrations, and the relative importance of each metabolic reaction is unknown. This must be kept in mind when interpreting the data. However, the gene expression data provide significant information in terms of highlighting the reactions that are most likely to be relevant for the observed differences in metabolic pattern. Hypotheses generated on the basis of microarray data should be evaluated by tracking the flux of metabolites through the different pathways.

Comparing our data with pre-existing studies of choline metabolism in cultured cells and *in vivo *models with data from human biopsies, we suggest that primary tumor xenografts are more relevant model systems than cell cultures with respect to investigation of metabolic profiles in different breast cancer subtypes, and may be a better approach to studies of therapeutic efficacy in the different breast cancer subtypes. As the choline metabolite profile of the xenograft models used in the study appear representative of basal-like and luminal-like human breast cancer, the models are considered valuable tools for testing of targeted drugs and for monitoring response to treatment in these subtypes of breast cancer.

## Conclusions

HR MAS MRS analyses of a basal-like and a luminal-like xenograft model demonstrated significant differences in choline metabolite concentrations. In the more aggressive basal-like tumor, GPC concentrations were higher than PCho concentrations, whereas this pattern was reversed in the luminal-like model. Glycine concentration was also significantly higher in the basal-like model. These differences could at least in part be explained by lower choline kinase expression and increased PtdCho degradation in the basal-like model. The gene expression data also suggested a possible shift in metabolic flux from PtdCho synthesis to glycine formation in the basal-like model. The choline metabolism pattern in the xenografts corresponded well with spectra from tissue samples from triple negative and ER+/PgR+ human breast carcinomas, suggesting that the basal-like and luminal-like xenografts may be a relevant model system for studies of choline metabolism in these two subtypes of human breast cancer.

## Competing interests

The authors declare that they have no competing interests.

## Authors' contributions

SAM conceived and designed the study, performed the HR MAS MRS analysis, performed the histopathological analysis, interpreted the data and wrote the manuscript. EB performed the gene expression analysis and interpreted the microarray data. EMH performed the HR MAS MRS analysis. EL carried out the *in vivo *experiments. BS established the HR MAS MRS protocol and supervised the analyses. ALBD contributed with expertise in molecular biology techniques. OE designed and coordinated the *in vivo *experiments. GMM participated in design and coordination of the study. ISG designed and coordinated the study and gave final approval of the manuscript. All co-authors critically revised the manuscript and approved the final version.

## Pre-publication history

The pre-publication history for this paper can be accessed here:

http://www.biomedcentral.com/1471-2407/10/433/prepub

## Supplementary Material

Additional file 1 **Differential gene expression**. Excel spreadsheet containing results from the differential gene expression analysis of the 119 investigated genes. Click here for file

## References

[B1] Perou CM Sorlie T Eisen MB van de RM Jeffrey SS Rees CA Molecular portraits of human breast tumours Nature 2000 406 747 752 10.1038/35021093 10963602

[B2] Sorlie T Perou CM Tibshirani R Aas T Geisler S Johnsen H Gene expression patterns of breast carcinomas distinguish tumor subclasses with clinical implications Proc Natl Acad Sci USA 2001 98 10869 10874 10.1073/pnas.191367098 11553815 PMC58566

[B3] Sorlie T Tibshirani R Parker J Hastie T Marron JS Nobel A Repeated observation of breast tumor subtypes in independent gene expression data sets Proc Natl Acad Sci USA 2003 100 8418 8423 10.1073/pnas.0932692100 12829800 PMC166244

[B4] Calza S Hall P Auer G Bjohle J Klaar S Kronenwett U Intrinsic molecular signature of breast cancer in a population-based cohort of 412 patients Breast Cancer Res 2006 8 R34 10.1186/bcr1517 16846532 PMC1779468

[B5] Kurebayashi J Moriya T Ishida T Hirakawa H Kurosumi M Akiyama F The prevalence of intrinsic subtypes and prognosis in breast cancer patients of different races Breast 2007 16 Suppl 2 S72 S77 10.1016/j.breast.2007.07.017 17714947

[B6] Mullins M Perreard L Quackenbush JF Gauthier N Bayer S Ellis M Agreement in breast cancer classification between microarray and quantitative reverse transcription PCR from fresh-frozen and formalin-fixed, paraffin-embedded tissues Clin Chem 2007 53 1273 1279 10.1373/clinchem.2006.083725 17525107

[B7] Sorlie T Wang Y Xiao C Johnsen H Naume B Samaha RR Distinct molecular mechanisms underlying clinically relevant subtypes of breast cancer: gene expression analyses across three different platforms BMC Genomics 2006 7 127 10.1186/1471-2164-7-127 16729877 PMC1489944

[B8] Stadler ZK Come SE Review of gene-expression profiling and its clinical use in breast cancer Crit Rev Oncol Hematol 2009 69 1 11 10.1016/j.critrevonc.2008.05.004 18614375

[B9] Banez-Coronel M de Molina AR Rodriguez-Gonzalez A Sarmentero J Ramos MA Garcia-Cabezas MA Choline kinase alpha depletion selectively kills tumoral cells Curr Cancer Drug Targets 2008 8 709 719 10.2174/156800908786733432 19075594

[B10] Hernandez-Alcoceba R Fernandez F Lacal JC In vivo antitumor activity of choline kinase inhibitors: a novel target for anticancer drug discovery Cancer Res 1999 59 3112 3118 10397253

[B11] Lacal JC Choline kinase: a novel target for antitumor drugs IDrugs 2001 4 419 426 16015482

[B12] Rodriguez-Gonzalez A Ramirez de MA Fernandez F Ramos MA del Carmen NM Campos J Inhibition of choline kinase as a specific cytotoxic strategy in oncogene-transformed cells Oncogene 2003 22 8803 8812 10.1038/sj.onc.1207062 14654777

[B13] Rodriguez-Gonzalez A Ramirez de MA Banez-Coronel M Megias D Lacal JC Inhibition of choline kinase renders a highly selective cytotoxic effect in tumour cells through a mitochondrial independent mechanism Int J Oncol 2005 26 999 1008 15753995

[B14] Tozaki M Proton MR spectroscopy of the breast Breast Cancer 2008 15 218 223 10.1007/s12282-008-0048-x 18443899

[B15] Aboagye EO Bhujwalla ZM Malignant transformation alters membrane choline phospholipid metabolism of human mammary epithelial cells Cancer Res 1999 59 80 84 9892190

[B16] Eliyahu G Kreizman T Degani H Phosphocholine as a biomarker of breast cancer: molecular and biochemical studies Int J Cancer 2007 120 1721 1730 10.1002/ijc.22293 17236204

[B17] Glunde K Jie C Bhujwalla ZM Molecular causes of the aberrant choline phospholipid metabolism in breast cancer Cancer Res 2004 64 4270 4276 10.1158/0008-5472.CAN-03-3829 15205341

[B18] Gillies RJ Morse DL In vivo magnetic resonance spectroscopy in cancer Annu Rev Biomed Eng 2005 7 287 326 10.1146/annurev.bioeng.7.060804.100411 16004573

[B19] Mackinnon WB Barry PA Malycha PL Gillett DJ Russell P Lean CL Fine-needle biopsy specimens of benign breast lesions distinguished from invasive cancer ex vivo with proton MR spectroscopy Radiology 1997 204 661 666 9280241 10.1148/radiology.204.3.9280241

[B20] Negendank W Studies of human tumors by MRS: a review NMR Biomed 1992 5 303 324 1333263 10.1002/nbm.1940050518

[B21] Sitter B Lundgren S Bathen TF Halgunset J Fjosne HE Gribbestad IS Comparison of HR MAS MR spectroscopic profiles of breast cancer tissue with clinical parameters NMR Biomed 2006 19 30 40 10.1002/nbm.992 16229059

[B22] Meisamy S Bolan PJ Baker EH Bliss RL Gulbahce E Everson LI Neoadjuvant chemotherapy of locally advanced breast cancer: predicting response with in vivo (1)H MR spectroscopy--a pilot study at 4 T Radiology 2004 233 424 431 10.1148/radiol.2332031285 15516615

[B23] Morse DL Raghunand N Sadarangani P Murthi S Job C Day S Response of choline metabolites to docetaxel therapy is quantified in vivo by localized (31)P MRS of human breast cancer xenografts and in vitro by high-resolution (31)P NMR spectroscopy of cell extracts Magn Reson Med 2007 58 270 280 10.1002/mrm.21333 17654590

[B24] Neeman M Eldar H Rushkin E Degani H Chemotherapy-induced changes in the energetics of human breast cancer cells; 31P- and 13C-NMR studies Biochim Biophys Acta 1990 1052 255 263 10.1016/0167-4889(90)90219-4 2334736

[B25] Glunde K Jie C Bhujwalla ZM Mechanisms of indomethacin-induced alterations in the choline phospholipid metabolism of breast cancer cells Neoplasia 2006 8 758 771 10.1593/neo.06187 16984733 PMC1584299

[B26] Morse DL Carroll D Day S Gray H Sadarangani P Murthi S Characterization of breast cancers and therapy response by MRS and quantitative gene expression profiling in the choline pathway NMR Biomed 2009 22 114 127 10.1002/nbm.1318 19016452 PMC4130559

[B27] Katz-Brull R Seger D Rivenson-Segal D Rushkin E Degani H Metabolic markers of breast cancer: enhanced choline metabolism and reduced choline-ether-phospholipid synthesis Cancer Res 2002 62 1966 1970 11929812

[B28] Vargo-Gogola T Rosen JM Modelling breast cancer: one size does not fit all Nat Rev Cancer 2007 7 659 672 10.1038/nrc2193 17721431

[B29] Bergamaschi A Hjortland GO Triulzi T Sorlie T Johnsen H Ree AH Molecular profiling and characterization of luminal-like and basal-like in vivo breast cancer xenograft models Mol Oncol 2009 3 469 482 10.1016/j.molonc.2009.07.003 19713161 PMC5527532

[B30] Sitter B Sonnewald U Spraul M Fjosne HE Gribbestad IS High-resolution magic angle spinning MRS of breast cancer tissue NMR Biomed 2002 15 327 337 10.1002/nbm.775 12203224

[B31] Sitter B Bathen TF Singstad TE Fjosne HE Lundgren S Halgunset J Quantification of metabolites in breast cancer patients with different clinical prognosis using HR MAS MR spectroscopy NMR Biomed 2010 23 424 431 10.1002/nbm.1478 20101607

[B32] Smyth GK Linear models and empirical bayes methods for assessing differential expression in microarray experiments Stat Appl Genet Mol Biol 2004 3 Article3 16646809 10.2202/1544-6115.1027

[B33] Kanehisa M Goto S KEGG: kyoto encyclopedia of genes and genomes Nucleic Acids Res 2000 28 27 30 10.1093/nar/28.1.27 10592173 PMC102409

[B34] Katz-Brull R Margalit R Degani H Differential routing of choline in implanted breast cancer and normal organs Magn Reson Med 2001 46 31 38 10.1002/mrm.1157 11443708

[B35] Michel V Yuan Z Ramsubir S Bakovic M Choline transport for phospholipid synthesis Exp Biol Med (Maywood) 2006 231 490 504 16636297 10.1177/153537020623100503

[B36] Gallazzini M Ferraris JD Burg MB GDPD5 is a glycerophosphocholine phosphodiesterase that osmotically regulates the osmoprotective organic osmolyte GPC Proc Natl Acad Sci USA 2008 105 11026 11031 10.1073/pnas.0805496105 18667693 PMC2504799

[B37] Kreike B van KM Horlings H Weigelt B Peterse H Bartelink H Gene expression profiling and histopathological characterization of triple-negative/basal-like breast carcinomas Breast Cancer Res 2007 9 R65 10.1186/bcr1771 17910759 PMC2242660

[B38] Sorlie T Molecular portraits of breast cancer: tumour subtypes as distinct disease entities Eur J Cancer 2004 40 2667 2675 10.1016/j.ejca.2004.08.021 15571950

[B39] Bhargava R Striebel J Beriwal S Flickinger JC Onisko A Ahrendt G Prevalence, morphologic features and proliferation indices of breast carcinoma molecular classes using immunohistochemical surrogate markers Int J Clin Exp Pathol 2009 2 444 455 19294003 PMC2655155

[B40] Katz-Brull R Degani H Kinetics of choline transport and phosphorylation in human breast cancer cells; NMR application of the zero trans method Anticancer Res 1996 16 1375 1380 8694504

[B41] Ramirez de MA Banez-Coronel M Gutierrez R Rodriguez-Gonzalez A Olmeda D Megias D Choline kinase activation is a critical requirement for the proliferation of primary human mammary epithelial cells and breast tumor progression Cancer Res 2004 64 6732 6739 10.1158/0008-5472.CAN-04-0489 15374991

[B42] Gribbestad IS Petersen SB Fjosne HE Kvinnsland S Krane J 1H NMR spectroscopic characterization of perchloric acid extracts from breast carcinomas and non-involved breast tissue NMR Biomed 1994 7 181 194 10.1002/nbm.1940070405 7946996

[B43] Kvistad KA Bakken IJ Gribbestad IS Ehrnholm B Lundgren S Fjosne HE Characterization of neoplastic and normal human breast tissues with in vivo (1)H MR spectroscopy J Magn Reson Imaging 1999 10 159 164 10.1002/(SICI)1522-2586(199908)10:2<159::AID-JMRI8>3.0.CO;2-0 10441019

[B44] Eliyahu G Maril N Margalit R Degani H Choline Metabolism in Breast Cancer; The Influence of the Microenvironmental conditions [Abstract] Proc Intl Soc Mag Reson Med 2007 15

[B45] Giskeodegard GF Grinde MT Sitter B Axelson DE Lundgren S Fjosne HE Multivariate modeling and prediction of breast cancer prognostic factors using MR metabolomics J Proteome Res 2010 9 972 979 10.1021/pr9008783 19994911

[B46] Mori N Glunde K Takagi T Xiong L Wides F Bhujwalla Z Tumor microenvironmental alterations of lipid metabolism detected by comparing cancer cells with tumors [Abstract] Proc Intl Soc Mag Reson Med 2009 17 2310

